# The Molecular Effects of BDNF Synthesis on Skeletal Muscle: A Mini-Review

**DOI:** 10.3389/fphys.2022.934714

**Published:** 2022-07-06

**Authors:** I. Rentería, P. C. García-Suárez, A. C. Fry, J. Moncada-Jiménez, J. P. Machado-Parra, B. M. Antunes, A. Jiménez-Maldonado

**Affiliations:** ^1^ Facultad de Deportes, Universidad Autónoma de Baja California, Ensenada, Mexico; ^2^ Department of Health, Sports and Exercise Sciences, University of Kansas, Lawrence, KS, United States; ^3^ Human Movement Sciences Research Center (CIMOHU), University of Costa Rica, San José, Costa Rica

**Keywords:** neurotrophin, BDNF, skeletal muscle, myokine, exercise, physical activity

## Abstract

The brain-derived neurotrophic factor (BDNF) is a member of the nerve growth factor family which is generated mainly by the brain. Its main role involve synaptic modulation, neurogenesis, neuron survival, immune regulation, myocardial contraction, and angiogenesis in the brain. Together with the encephalon, some peripheral tissues synthesize BDNF like skeletal muscle. On this tissue, this neurotrophin participates on cellular mechanisms related to muscle function maintenance and plasticity as reported on recent scientific works. Moreover, during exercise stimuli the BDNF contributes directly to strengthening neuromuscular junctions, muscle regeneration, insulin-regulated glucose uptake and *β*-oxidation processes in muscle tissue. Given its vital relevance on many physiological mechanisms, the current mini-review focuses on discussing up-to-date knowledge about BDNF production in skeletal muscle and how this neurotrophin impacts skeletal muscle biology.

## Introduction

The brain-derived neurotrophic factor (BDNF) is a member of the nerve growth factor family mainly synthesized by neurons as a pre-protein (proBDNF, 32 kDa) (Conner et al., 1997; Mowla et al., 2001; Cunha et al., 2010). Two activity-dependent secretory vesicles derive from the proBDNF translocation *via* the Golgi apparatus into the trans-Golgi network. The first protein involves proteolytic processing to develop a mature protein (BDNF or mBDNF) (14 kDa); while the second protein fate implicates the secretion of proBDNF (see (Lessmann et al., 2003; Leßmann and Brigadski, 2009; Cunha et al., 2010) for an explanation about the molecular mechanism of sorting BDNF). Once released, the mature neurotrophin binds to a tyrosine kinase B receptor (TrkB). This BDNF-TrkB interaction induces dimerization of TrkBs and generates auto-phosphorylation that releases a signal transduction pathway (Blum and Konnerth, 2005; Sasi et al., 2017).

BDNF regulates cellular responses, such as synapses modulation, neurogenesis, axonal growth, and neuron survival (Ghosh et al., 1994; Tyler and Pozzo-Miller, 2001). Furthermore, peripheral BDNF is involved in immune regulation, myocardial contraction, and angiogenesis (Hong et al., 2014; Yu et al., 2016; Lee et al., 2018). These responses could be explained *via* the autocrine and paracrine synthesis mechanisms of BDNF by non-neuronal tissues (e.g., adipose tissue, endothelial cells, and skeletal muscle) (Liem et al., 2001; Aid et al., 2007). Given the pivotal relevance of BDNF on several physiological mechanisms, the current mini-review focuses on discussing up-to-date knowledge about BDNF production in skeletal muscle and how this neurotrophin impacts skeletal muscle biology.

## The *Bdnf* Gene Structure and BDNF Expression in Skeletal Muscle

The Bdnf gene structure in rats was early characterized by Timmusk et al. (Timmusk et al., 1993), who identified five exons (I, II, III and IV, V). The first four (I-IV) were labeled as 5′ noncoding exons and the fifth (V) exon as a common 3′ coding exon. However, given the advances in the field in the last 20 years, rodents’ Bdnf gene structure and nomenclature have changed (Aid et al., 2007; Pruunsild et al., 2007). Currently, it is known that Bdnf gene in rodents involves nine exons, the first eight (I-VIII) are 5′ noncoding exons, and there is only one coding 3’exon (IX) ((Aid et al., 2007; Pruunsild et al., 2007; Cunha et al., 2010; Nair and Wong-Riley, 2016) for *Bdnf* gene structure details). These exons’ expressions generate 24 mRNA Bdnf transcripts specific to neuronal and non-neuronal tissues. Nevertheless, all the Bdnf transcripts encode for the same mature BDNF protein (Cunha et al., 2010). In humans, the Bdnf gene structure resides in chromosome 11, and span’s on ≈70 kb (base pairs) involving 11 exons (I-IX, plus Vh VIIIh) (Pruunsild et al., 2007; Cattaneo et al., 2016). Although, such rodents, the IX promoter contains the encoding sequence (Pruunsild et al., 2007), the splicing of exons generates 20 mRNA Bdnf transcripts (Cattaneo et al., 2016). However, likewise rodents, only a mature BDNF protein is produced (Pruunsild et al., 2007).

The IV promoter (early labeled as promoter III in Timmusk´s nomenclature) is common (i.e., homologous) in rodents and humans (Pruunsild et al., 2007), and it is expressed dependent on activity (Tao et al., 2002). Concretely, basic research using neurons indicates that intracellular messengers such as Ca^++^ and cyclic adenosine monophosphate (cAMP) indirectly activate the IV transcription (Tao et al., 1998, 2002). Furthermore, this transcript is expressed in skeletal muscle (Aid et al., 2007); therefore, authors have suggested a similar mechanism in skeletal muscle that explains the IV promoter expression.

Early studies on electrical stimulation of the sciatic nerve aided to understand the effect of muscle activity on *Bdnf* expression (Park et al., 2004). Park et al. used a dose-response model and found that higher frequency stimulation (1 ms/40 Hz/30 min) generated the more significant expression of Bdnf mRNA and BDNF protein in the *soleus* and *gastrocnemius* muscles of healthy rats (Park et al., 2004). The authors suggested that a continuous electric stimulation generates muscle fiber damage, and consequently, this controlled injury enhanced the levels of *Bdnf* mRNA, and consequently BDNF protein in skeletal muscle (Park et al., 2004). The same model was used in diabetic rats (Copray et al., 2000); in this work, the muscle damage found was considered the main factor inducing Bdnf expression in skeletal muscle. The association supported this hypothesis as found among the Bdnf mRNA levels in muscle and plasmatic creatine kinase activity, a muscle damage biomarker (Copray et al., 2000). The function of BDNF synthesized during muscle injury involves neurotrophin participation in the muscle repair signaling cascade (Lian et al., 1998) and also in maintaining muscle innervation (Copray et al., 2000).

Even though we broadly discuss how muscle damage and injury are contributors to *Bdnf* expression and BDNF synthesis in muscle, these are not unique constituents of these molecular processes. Locomotor activity itself is a substantial provocation for the making of this neurotrophin in skeletal muscle(Gómez-Pinilla et al., 2001). In line with this, physical exercise (PE) performed on a wheel running (voluntary) and treadmill (forced) are an efficient stimulus for eliciting *Bdnf* expression and BDNF synthesis in skeletal muscle in rodent (Gómez-Pinilla et al., 2001, 2002; Cuppini et al., 2007; Ogborn and Gardiner, 2010; Jiménez-Maldonado et al., 2016; Zhang et al., 2019) (Table 1). Moreover, the studies performed in treadmill concluded that muscle phenotype is a modulatory variable for the BDNF response on PE (Ogborn and Gardiner, 2010; Jiménez-Maldonado et al., 2016). Opposite, the molecular mechanism stimulated by voluntary wheel running responsible for inducing eliciting *Bdnf* expression and BDNF synthesis in muscle has not been determined yet. Nevertheless, it has been hypothesized that the neurotrophin produced in the *soleus* muscle during voluntary PE underwent retrograde transport to improve the electro-physiologic properties of motor neurons (Gómez-Pinilla et al., 2001). Opposite to slow muscle, there is no report aimed at determining the effect of the voluntary wheel running on the BDNF synthesis in fast muscle. Therefore, scientific studies are needed to determine the effects of voluntary PE models on specific muscle fiber types.

**TABLE 1:** Summary of studies on BDNF synthesis in skeletal muscle regulated by the physical exercise.

Authors	Species	Exercise Model (Protocol)	Muscle Studied	Main Finding
Goméz-Pinilla et al. (2001)	Rats	Treadmill exercise (5 consecutive days: 30 min/session at 27 m/min, 3% incline)	*Soleus*	The BDNF protein levels were increased ≈130% of control
Goméz-Pinilla et al. (2001)	Rats	Treadmill exercise (1 day of exercise: 30 min/session at 27 m/min, 3% incline)	*Soleus*	The acute exercise did not modify the mRNA *Bdnf* levels
Goméz-Pinilla et al. (2002)	Rats	Running wheel (3 and 7 days of voluntary exercise). Every day, 100 g of resistance were added to wheels	*Soleus*	The *Bdnf* mRNA and BDNF proteins levels were significantly higher at 3 and 7 days compared with control
Cuppini et al. (2007)	Rats	Treadmill exercise (5 consecutive days: 30 min/session: intermittent exercise: 5 min of running exercise following constant acceleration (27 m/min maximum speed), 5 min of rest)	*Soleus*	The Bdnf mRNA levels increased transitory after 2 h of finished the repetitive exercise program. Proteins BDNF levels increased at 2 and 24 h after completing the last session of the short-program
Cuppini et al. (2007)	Rats	Treadmill exercise (acute exercise: 30 min/session: intermittent exercise: 5 min of running exercise following constant acceleration (27 m/min maximum speed), 5 min of rest)	*Soleus*	The Bdnf mRNA levels increased significantly only after 24 and 48 h of finished the exercise. Whereas the proteins BDNF levels increased at 48 and 72 h after completed the exercise
Matthews et al. (2009)	Human	Bicycle exercise (120 min at 60% of VO_2max_)	*Vastus lateralis*	The *Bdnf* mRNA levels were unmodified by exercise. Contrary, 24 h after finished the acute exercise, the BDNF protein levels increased significantly
Ogborn and Gardiner. (2010)	Rats	Treadmill exercise (5 consecutive days: 30 min/session at a speed of 27 m/min 3% incline)	Medial *gastrocnemius* and *soleus*	In the *soleus* the *Bdnf* mRNA levels increase more than 180% compared with control gene. Opposite, in the gastrocnemius, the *Bdnf* mRNA levels does not changed
Ogborn and Gardiner. (2010)	Rats	Treadmill exercise (10 consecutive days: 30 min/session at a speed of 20 m/min. In the final 5 days of this protocol the incline of the treadmill was increased to 5%)	Medial *gastrocnemius* and *soleus*	In the *soleus* and *gastrocnemius*, the *Bdnf* mRNA levels were unmodified by exercise. Likewise, the BDNF protein levels did not change after exercise
Jiménez-Maldonado et al. (2016)	Rats	Treadmill exercise (24 exercise sessions: 10 min warm-up; and 60 min of exercise running at 22 m/min for the MIT and 28 m/min for the HIT group). The sessions ended with a 10-min cool-down (18 m/min)	*Soleus* and *plantaris*	The *Bdnf* mRNA levels in the fast muscle were not modified by the long-term exercise. Contrary, in the slow muscle, the HIT protocol increased more than 300% *Bdnf* mRNA levels with respect to control
				The BDNF protein levels were significantly reduced by HIT. Opposite, in the soleus muscle, the BDNF protein levels not statistically modified through the treatment
Walsh et al. (2014)	Human	Bicycle exercise (3 sessions of HIIE at 73, 100, or 133% of their VO_2peak_)	*Vastus lateralis*	The *Bdnf* mRNA levels were unmodified by the treatment
de Assis et al. (2021)	Human	Graded exercise test (Bruce treadmill protocol)	*Vastus lateralis*	The *Bdnf* mRNA levels reduced 44% immediately finished the maximum effort test. This response was independent of the carrier polymorphism “Val66Met”

Note: BDNF, Brain-derived neurotrophic factor; MIT, moderate intensity training; HIIE, High-intensity interval exercise; HIT, high intensity training; VO_2max_, Maximal oxygen uptake; VO_2peak_, Peak oxygen uptake.

Contrary with the voluntary exercise,, it has been established that forced running treadmill exercise enhances Bdnf transcription and BDNF synthesis in fast and slow muscle fibers (Gómez-Pinilla et al., 2002; Cuppini et al., 2007; Ogborn and Gardiner, 2010; Jiménez-Maldonado et al., 2016). In detail, slow muscle fibers (e.g., *soleus*) express higher Bdnf mRNA levels than fast phenotype muscle fibers (e.g., *gastrocnemius*, *plantaris*) (Cuppini et al., 2007; Ogborn and Gardiner, 2010; Jiménez-Maldonado et al., 2016). Considering that the IV exon expresses in skeletal muscle (Aid et al., 2007) and activated *via* Ca^++^ influx (Tao et al., 1998, 2002), is expected that fast-twitch fibers with a greater cytoplasmic Ca^++^ content during muscle contraction or electrical muscle stimulation is larger than slow-muscle fibers (Baylor and Hollingworth, 2012). However, evidence shows that Bdnf mRNA levels in slow-twitch fibers is higher in contrast to fast muscle. This is an unexpected response. Henceforth, authors have indicated that the mRNA stability is more relevant than the Ca^++^ activation to explain the differences of Bdnf mRNA levels after forced treadmill running in slow and fast muscle fibers (Jiménez-Maldonado et al., 2016). Concretely, a possible hint could be found on protein kinase C (PKC), a molecule with more catalytic activity in the *soleus* than in fast muscles during PE (Krisan et al., 2004), could bring stability to Bdnf mRNA, a phenomenon observed in neurons (Zafra et al., 1992). The potential mechanism includes the phosphorylation and inhibition of the coactivator-associated arginine methyltransferase 1 (CARM1). One inactivated, CARM1 reduced the methylation effect on HuD, a mRNA binding protein which stabilized the Bdnf mRNA (Lim and Alkon, 2012).

Opposite with data reported in regard with the *Bdnf* expression in muscle after the treadmill running intervention, there are inconsistent findings regarding the neurotrophin concentration in this scenario. For example, some researchers found high BDNF protein levels after exercise in slow muscle fibers (e.g., *soleus*) (Cuppini et al., 2007); others reported lower neurotrophin levels in *soleus* muscle in exercised rats compared to sedentary animals (Jiménez-Maldonado et al., 2016). Several factors can explain the non-concordant data; one is the training intervention length. In this sense, a study used acute PE and five consecutive days of PE (Cuppini et al., 2007). Instead, Jiménez-Maldonado (Jiménez-Maldonado et al., 2016) involved 24 PE sessions. The PE intervention length has always been considered a variable with a substantial role in BDNF production (Gomez-Pinilla et al., 2002). Furthermore, the training modality was also different; concretely, intermittent exercise training was used by Cupinni’s group, and a continuous PE ruining protocol was used in the study by Jiménez-Maldonado et al. (Jiménez-Maldonado et al., 2016). In this sense, intermittent exercise has been identified as a stronger treatment to increase BDNF (Marquez et al., 2015; García-Suárez et al., 2021). Moreover, it has been suggested that the reduction of BDNF protein levels observed in the slow muscle (i.e., *soleus*) after chronic treadmill running resulted from a retrograde transport activated by PE (Jiménez-Maldonado et al., 2016). This hypothesis also was suggested for fast muscle (i.e., *plantaris*) (Jiménez-Maldonado et al., 2016).

Similar to rodents, the effect of PE on neurotrophin synthesis in human skeletal muscle has also been studied (Matthews et al., 2009; Walsh et al., 2014; de Assis et al., 2021). Nevertheless, in humans, only the acute PE paradigm has been studied. The available evidence shows that the mRNA Bdnf levels were unmodified by high-intensity interval training or moderate cycling exercise (Walsh et al., 2014) (Matthews et al., 2009); . This response can result from a delayed muscle response in gene expression (Louis et al., 2007; Walsh et al., 2014). Other evidence suggests a significant reduction of mRNA Bdnf levels after a graded and maximal exercise test (de Assis et al., 2021). Acute metabolic stress caused by exhaustive PE was the main factor to explain the decay in Bdnf expression (de Assis et al., 2021). Contrary to the mRNA´s responses, the protein levels of BDNF were transiently increased by the acute moderate PE (Matthews et al., 2009); however, this effect was observed until 24-h after finishing the moderate cycling exercise. In regard with their results, the authors indicated that the neurotrophin synthesized during the exercise acts in an autocrine and paracrine manner within the skeletal muscle (Matthews et al., 2009).

## Effects of Muscular BDNF on Neuromuscular Junction Physiology

Neuromuscular junctions (NMJ) are essential for muscular activity and consequently for human life preservation. NMJ consists in the release of acetylcholine (Ach) from the presynaptic moto-neuron to postsynaptic muscle endplate (Li et al., 2018; Rodríguez Cruz et al., 2020), activating the muscular Ach receptors (AChRs) provoking the muscle contraction (Li et al., 2018). The participation of muscle BDNF on NMJ function is a widely studied phenomenon (Gonzalez and Collins, 1997; Gonzalez et al., 1999; Garcia et al., 2010a, 2010b; Dorsey et al., 2012). In detail, a classic work demonstrated that muscle BDNF participates in the survival process of motor neurons after sciatic nerve transection (Funakoshi et al., 1993). This action is performed by a retrograde transport induced by the nerve injury (Funakoshi et al., 1993; Dupont-Versteegden et al., 2004; Omura et al., 2005). Furthermore, it was also identified that muscle-derived BDNF increases the motor neuron excitability; this effect was generated by reducing the rheobase and total cell capacitance (Gonzalez and Collins, 1997). In addition, recent evidence found that BDNF synthesized by the muscle during contraction, strengthened the synaptic function, an affect induced by the interaction among the neurotrophin and TrkB receptor. Once activated, TrkB enhances the functions of the presynaptic protein kinase C family (cPKCα, cPKCβI, and cPKCε) (Obis et al., 2015; Hurtado et al., 2017). After the activation, PKCs deliver the signaling pathway that enhances the synaptic vesicle fusion and neurotransmitter release (Obis et al., 2015; Hurtado et al., 2017).

Besides strengthening the ACh secretion at the motor endplate, muscle-derived BDNF attenuates the synapses elimination in postnatal mice; this action is partially regulated by metalloprotease activity (Shawn Je et al., 2013). Concretely, the metalloprotease converts proBDNF to mBDNF, and consequently, the mBDNF is released and interacts with TrkB in the nerve terminals to facilitate muscle innervation (Shawn Je et al., 2013). Finally, other studies indirectly highlighted the BDNF participation in the NMJ (Kulakowski et al., 2011). Kulawkiski et al. (Kulakowski et al., 2011) studied heterozygous B6.129S2-Ntrk2tmlBbd/J (TrkB^+/−^) mice; a model used to hinder the TrkB full-length activity in the *soleus* NMJ. The authors found disruptions in the AchR cluster in the pre and postsynaptic regions. Additionally, with the morphological changes, the blockage of the BDNF receptor hindered the neuromuscular transmission, resulting in higher muscle fatigue (Kulakowski et al., 2011). Likewise, the maximal muscle force production, specific force, and fiber cross-sectional area were reduced in the heterozygote mice (Kulakowski et al., 2011). These data indirectly support the BDNF participation to stabilize the NMJ in the slow muscle fiber phenotype.

## Effects of Muscular BDNF on Myogenesis

Myogenesis is a biological process focused on building skeletal muscle. It is present in several periods during growth and development, and as can be supposed, in each stage, the myogenesis is regulated by several factors and conditions. Specifically, during the embryonic phase (EP), the early myogenesis comes from the paraxial mesoderm; this layer provides the precursor cells to generate somites (Chal and Pourquié, 2017). The somites (i.e., dermomyotome) receive signals from adjacent tissues that lead to the myogenic lineage cells (Pax3+ve), which express the primary myogenic regulator factors (MRF) Myf5 and MyoD (see (Chargé and Rudnicki, 2004; Tajbakhsh, 2009; Chal and Pourquié, 2017) for an in-depth explanation). After that, Myf5 and MyoD committed to the satellite cells to generate myoblast (Pax7+ve), cells that express secondary MRF such as myogenin and MRF4S (fetal state) (Bentzinger et al., 2012). The secondary MRFs lead to myoblast’s final differentiation into myocytes, which are fused with the muscle myofiber to generate a multinucleated tissue (perinatal state) (Chargé and Rudnicki, 2004). It is worth indicating that the skeletal muscle has quiescent satellite cells localized under the basal-lamina; these cells will participate in the adult myogenesis (Mauro, 1961; Chargé and Rudnicki, 2004; Chal and Pourquié, 2017; Hernández-Hernández et al., 2017). Furthermore, the stem cell dynamics are still present at the post-natal phase; particularly during adulthood, muscle injury is one of the main factors responsible for activating the quiescent satellite cells (Jejurikar and Kuzon, 2003; Chargé and Rudnicki, 2004; Karalaki et al., 2009). In detail, muscle remodeling implicates two phases, degeneration and regeneration (Chargé and Rudnicki, 2004; Karalaki et al., 2009). The first phase begins in the early post-exercise hours, neutrophils dominate the inflammatory cell profile, acting to clear cellular debris and propagating the inflammatory response by cytokine secretion. Mast cells also infiltrate muscle tissue, releasing histamine and chemoattractants. Between 4 and 24 h after muscle damage, pro-inflammatory macrophages invade muscle, secreting pro-inflammatory cytokines, phagocytizing damaged tissue (Peake et al., 2017). On the other hand, the regeneration phase involves differentiation, proliferation, and fusion of myogenic cells (i.e., satellite cells) (Chargé and Rudnicki, 2004; Karalaki et al., 2009). The scientific evidence indicates that muscle BDNF participates in the regeneration phase (Mousavi and Jasmin, 2006; Clow and Jasmin, 2010). By studying mice with depleted BDNF from skeletal muscle and whole BDNF^−/−^ mice, Clow’s et al. (Clow and Jasmin, 2010) work demonstrated that BDNF positively regulates the function of satellite cells during muscle regeneration. Coupled with these findings, others reported that BDNF is a relevant factor in maintaining a pool of muscular progenitor cells in the muscle (Mousavi and Jasmin, 2006). The previous information highlights the relevance of BDNF on skeletal muscle regeneration.

## Metabolic Impact of Muscular BDNF

The effect of BDNF on neuronal networking and muscle-nerve communication is well-characterized. However, in addition to its classical role, the evidence supports the participation of BDNF in metabolic processes (Nakagawa et al., 2000; Suzuki et al., 2007; Fulgenzi et al., 2020). In the current section, we will focus on discussing the direct and indirect regulation of BDNF on the process linked with skeletal muscle metabolism. Recent evidence reported high BDNF and p-TrkB in the *soleus* muscle after eight weeks of moderate-intensity running exercise; these results were accompanied by a high p-AMPK and PGC-1α levels (Zhang et al., 2019). The findings brought the authors to emphasize the neurotrophin’s participation in the muscle adaptation to PE. The conclusion was supported by data showing that high p-AMPK levels enhanced skeletal muscle glucose uptake (Mu et al., 2001; Jørgensen et al., 2004, 2006). Moreover, AMPK activation facilitates the trans-sarcolemma uptake of long-chain fatty acid through the translocation of the fatty acid translocase FAT/CD36 (Luiken et al., 2003; Palanivel and Sweeney, 2005; Jørgensen et al., 2006) and fatty acid oxidation (Merrill et al., 1997; Jørgensen et al., 2006). The later process is mediated by phosphorylation and inactivation of acetyl-CoA carboxylase (ACC) and decreased malonyl-CoA levels (Merrill et al., 1997; Jørgensen et al., 2006). On the other hand, the PGC-1α is the master regulator for mitochondrial biogenesis (Puigserver et al., 1998; Wu et al., 1999). The previous information allows hypothesizing that the BDNF action in muscle could increase the mitochondrial mass and improve the aerobic metabolism of the macronutrients in the same cellular organ resulting in better exercise tolerance as reported before (Zhang et al., 2019). In addition to the results described above, others reported higher GLUT4 levels in the mice’s *gastrocnemius* after BDNF subcutaneous administration (14 days/20 mg/kg body mass) (Suwa et al., 2010). Furthermore, in concordance with the *in vivo* models, *in vitro* experiments (L6 and C2C12 myotubes) showed the participation of BDNF on the fatty acid oxidation (FAO), an effect induced through AMPK (Matthews et al., 2009; Yang et al., 2019). In the same sense, was uncovered that during fasting conditions (glucose-deprived medium) BDNF-muscle derived increases the synthesis of mitochondrial proteins (cytochrome c (Cyto c), succinate dehydrogenase (SDH), pyruvate dehydrogenase (PDH)] and mitochondrial DNA (mtDNA), and molecules linked with mitochondrial biogenesis (PGC-1α), those molecular adaptations were the main explanation about the larger FAO induced by the neurotrophin (Yang et al., 2019). The same study employing the transgenic mice (muscle-specific *Bdnf* knockout -MBKO- mice) model demonstrated that the BDNF-muscle derived reduced the energy metabolism leading to a bigger body weight and adipose tissue. Together with this, the authors also reported that impairing the BDNF-muscle derived action in the skeletal muscle, an accumulation of lipids in muscle is observed. The latter condition lead to development insulin resistance (Yang et al., 2019), it is worth to mention that the metabolic effects were observed in female mice, but not in male rodents, the authors suggest that the estrogen receptor function in muscle can be a key factor to explain the sexually dimorphic effect (Yang et al., 2019). Finally, employing the transgenic mice (BDNFMKO) model, the authors showed that BDNF promotes the transition from IIB into IIX fibers muscle, generating a more glycolytic profile muscle (Delezie et al., 2019). However, despite the promising findings, the molecular mechanism that linked the BDNF pathway with the metabolic proteins is not entirely elucidated (Zhang et al., 2019).

## Perspectives and conclusions

Early studies demonstrated the relevant participation of BDNF in neural plasticity; nonetheless, the continuous scientific work revealed novel peripheral functions for BDNF, such as immune system regulation and cardiovascular and metabolic processes. Even though skeletal muscle can produce BDNF by itself, its endocrinal role through BDNF is unclear.

**FIGURE 1:**
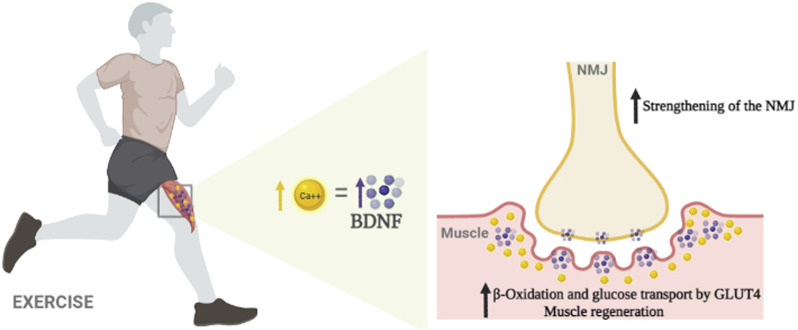
A schematic diagram showing the putative effects of the BDNF synthesized by the skeletal muscle during physical exercise. Ca++ seemingly regulates this process. Once produced, the neurotrophin works autocrine and paracrine to strengthen the NMJ. In addition, the BDNF participates indirectly in muscle regeneration at sustaining the pool of satellite cells. Finally, the neurotrophin enhances fat metabolism (β-oxidation) and facilitates glucose transport by increasing the GLUT4 transporter levels in the muscle.

On the other hand, the autocrine function of BDNF in muscle is solid; this neurotrophin regulates NMJ physiology and participates in the muscle’s metabolic flexibility control (e.g., fatty acid oxidation, increases glucose transporters content, glucose uptake facilitation), with the last having some promising findings. One hypothesis indicates BDNF’s participation in muscle plasticity following PE stimuli; furthermore, its role in muscle’s metabolic flexibility seems better understood in females than males in rodents (Figure 1).

Therefore, future studies are needed to clarify the participation of muscle-derived BDNF on metabolic flexibility in humans; while also elucidating the relevance of gender for the BDNF function. Besides, assessing how common metabolic disorders (e.g., obesity, metabolic syndrome, type 2 diabetes) modify the muscular neurotrophin synthesis during PE and prospect works addressing how aging mediates BDNF’s expression in skeletal muscle are encouraged.
